# Disability-related inequalities in lung cancer diagnostic pathways and outcomes: a population-based study in Northern Italy

**DOI:** 10.1093/eurpub/ckag205

**Published:** 2026-11-10

**Authors:** Flavia Pennisi, Carlotta Buzzoni, Federico Gervasi, Maria Teresa Greco, Carlo Signorelli, Antonio Giampiero Russo, Cristina Renzi

**Affiliations:** School of Medicine, University Vita-Salute San Raffaele, Milan, Italy; PhD National Programme in One Health Approaches to Infectious Diseases and Life Science Research, Department of Public Health, Experimental and Forensic Medicine, University of Pavia, Pavia, Italy; Epidemiology Unit, Agency for Health Protection of Milan, Milan, Italy; Epidemiology Unit, Agency for Health Protection of Milan, Milan, Italy; Epidemiology Unit, Agency for Health Protection of Milan, Milan, Italy; Epidemiology Unit, Agency for Health Protection of Milan, Milan, Italy; School of Medicine, University Vita-Salute San Raffaele, Milan, Italy; Epidemiology Unit, Agency for Health Protection of Milan, Milan, Italy; School of Medicine, University Vita-Salute San Raffaele, Milan, Italy; Research Department of Behavioural Science and Health, University College London, London, United Kingdom

## Abstract

People with a disability, representing one-quarter of the European population, can have difficulties accessing appropriate healthcare. Emergency presentation (EP) is a frequent and adverse route to lung cancer diagnosis, but evidence on diagnostic route and survival by disability status is limited. This study aims to assess the association between pre-existing disability and route to lung cancer diagnosis and 1-year survival. We conducted a population-based retrospective cohort study of incident lung cancer cases diagnosed in 2018–21 in Northern Italy. Using individually linked cancer registry and administrative healthcare data, we identified pre-existing disabilities through a validated algorithm based on the International Classification of Functioning, Disability and Health. Primary outcomes were route to diagnosis and 1-year all-cause mortality. Adjusted associations were estimated using multivariable logistic and Cox regression models. Among 9285 patients with lung cancer, 20.7% had at least one pre-existing impairment. EP occurred in 50.6% of patients with impairments versus 36.6% among those without. In adjusted analyses, impairments of mental functions (adjusted odds ratio [aOR] 1.67, 95% confidence interval [CI] 1.41–1.98), neuromusculoskeletal and movement-related impairments (aOR 1.29, 95% CI 1.04–1.61), and use of major prosthetic/orthotic devices (aOR 1.50, 95% CI 1.29–1.75) were associated with higher odds of EP, whereas sensory impairments were associated with lower odds. One-year mortality was higher for people with impairments of mental functions and prosthetic/orthotic device use. Pre-existing disability was associated with EP and 1-year mortality in lung cancer. Integrating functional impairment indicators into cancer surveillance and early diagnosis strategies may help identify high-risk groups and reduce inequalities in outcomes.

## Introduction

Lung cancer is the leading cause of cancer-related mortality worldwide. In Europe, lung cancer is responsible for more than 384 000 deaths annually, representing the leading cause of cancer-related mortality [1, 2]. In Italy, approximately 44 000 new lung cancer diagnoses were estimated in 2023, with 35 700 lung cancer deaths estimated in 2022 [3]. Lung cancer represents the leading cause of cancer-related mortality in men (24.0%) and the second in women (14.3%) [3].

People with disabilities, particularly those with severe mental illness, intellectual disabilities, or mobility impairments, have a higher risk of developing lung cancer due to behavioural and environmental risk factors. Evidence also indicates persistent disparities in healthcare access, including oncological care pathways [4] among people with disabilities, despite policies promoting inclusive healthcare [5]. Disparities have been further highlighted in recent years, particularly during the COVID-19 pandemic [6]. Disability, including physical, sensory, cognitive, and mental health conditions, affects ∼ 1.3 billion individuals globally [7], at least 135 million across the WHO European Region [8] and nearly one-quarter of the Italian population [9]. The disability prevalence increases markedly in older age groups, representing also the population strata most affected by cancer. Despite the potential relevance of these intersections, and despite formal healthcare rights to equitable access to diagnostic and oncological care for people with disabilities [7], population-based evidence examining the relationship between disability status, diagnostic pathways, and lung cancer outcomes remains scarce [10, 11].

Overall, population-level survival in lung cancer patients remains poor, despite substantial therapeutic advances over the past decade [12]. The 5-year net survival is 16% in men and 23% in women in Italy [13], which is in line with other European countries. Early diagnosis is essential for improving survival, however international data shows that up to 50% of lung cancer patients are diagnosed at advanced stage or following an emergency presentation (EP) [14], a diagnostic route consistently associated with poor outcomes [15] and healthcare disruptions. Reducing EP and advanced-stage diagnoses are key clinical and public health targets.

However, population-based evidence on EP for lung cancer in the European context remains scarce, with available data largely limited to England, where EP accounted for approximately one-third of all lung cancer diagnoses [16], and Denmark, where 28% of patients were diagnosed via unplanned admission [17]. In Italy, only one retrospective single-centre study has examined lung cancer patients diagnosed following EP, reporting advanced disease stage in the majority of cases, poor performance status, and suboptimal access to systemic treatment, with a median overall survival of 3.9 months [18]. A small number of studies on colorectal cancer have indicated a higher risk of EP among individuals with serious mental illness [19, 20]; however, no comparable evidence exists for other disability types or for lung cancer specifically.

The study aims to provide population-based evidence on routes to cancer diagnosis and variations in the risk of EP and survival by pre-existing disability, defined by the International Classification of Functioning, Disability and Health framework (ICF [21]), among patients diagnosed with lung cancer in Northern Italy, taking cancer-related and patient-level characteristics into account.

## Methods

### Study design and study population

We conducted a retrospective, population-based cohort study using data from the Milan Cancer Registry, linked with administrative healthcare databases from the Agency for Health Protection of Milan (ATS Milan), part of the Lombardy Regional Health System in Northern Italy. The study area comprises the provinces of Milan and Lodi, covering a resident population of ∼ 3.5 million individuals. Deterministic record linkage was performed through anonymized individual health-system beneficiary identifiers.

The study cohort included all incident cases of lung cancer diagnosed between 2018 and 2021 among residents of the ATS Milan area, classified according to the ICD-O-3.2 Classification World Health Organization (2019 [22]). The study was conducted in accordance with the Strengthening the Reporting of Observational Studies in Epidemiology guidelines [23].

### Data sources and variable definitions

The primary outcomes were route to diagnosis (RtD) and overall survival. RtD refers to the sequence of healthcare contacts leading to the diagnosis of lung cancer, as defined in previous international studies [14, 24]. As no single variable identifying the route to cancer diagnosis is available in Italian administrative healthcare data, RtD was inferred using a previously developed algorithm based on healthcare contacts preceding cancer diagnosis [25]. The algorithm integrates hospital discharge records, outpatient specialist records, and emergency department records and uses the temporal proximity between healthcare contacts and the date of cancer diagnosis to identify the most likely diagnostic route. Healthcare contacts occurring within 30 days before diagnosis were considered first; when no relevant contact was identified within this period, records from the preceding 6 months were examined. For the present study, diagnostic routes were classified into two mutually exclusive categories: EP and diagnosis following inpatient or outpatient care (IP/OP). A schematic representation of the RtD classification algorithm is provided in Supplementary Fig. S1.

The main exposure of interest was the presence of a pre-existing functional impairment, identified using an ICF-based algorithm developed and previously evaluated by ATS Milan [26] that integrates multiple healthcare, social-care, and administrative data sources. In the first step, diagnostic codes from healthcare and social service flows, including copayment exemptions, rehabilitation services, long-term residential care, and domiciliary assistance, were identified and mapped to the 2018 ICF [21]. Acute and traumatic conditions and chronic diseases not associated with disability-related complications were excluded during the development of the diagnostic-code set. The retained codes were assigned to the corresponding ICF body functions and structures and subsequently reviewed by a multidisciplinary clinical expert panel using a modified Delphi process [26]. In a second phase, healthcare administrative data were queried, including hospital discharge records and outpatient specialist visits, as well as data flows related to financial support provided by the Lombardy Region to individuals with disabilities and medical device registries, with the aim of identifying additional cases. A distinction was made between incontinence-related devices and the use of major prosthetic or orthotic devices, such as motor or limb prostheses. Each individual was classified according to the body structure and function affected by the impairment and could be assigned to one or more of eight impairment categories based on the ICF: mental functions; sensory functions and pain; phonatory and speech production functions; cardiovascular, respiratory, haematological, and immune systems; digestive, metabolic, and endocrine systems; genitourinary and reproductive systems; neuromusculoskeletal and movement-related functions; and skin and related structures. When classification according to the ICF framework was not feasible, but individuals were identified through prosthetic data flows or as beneficiaries of disability exemptions with attendance allowance, three additional categories were defined: major prosthetic or orthotic use; presence of a disability exemption with attendance allowance; and presence of incontinence-related devices. Methodological details of the identification algorithm have been reported elsewhere [26]. A schematic representation of the impairment identification and classification algorithm is provided in Supplementary Fig. S2. For the present study, impairment status was ascertained from all available administrative records preceding lung cancer diagnosis, without a fixed look-back period; only qualifying records documented before cancer diagnosis contributed to the definition of pre-existing impairment.

The ICF provides an internationally standardized framework for classifying functioning and disability, conceptualizing disability as a multidimensional construct encompassing impairments, activity limitations, and participation restrictions within a biopsychosocial model of health. This approach allowed us to operationalize pre-existing functional impairments within a standardized conceptual framework [26, 27]. To limit heterogeneity, only impairment categories with a prevalence of at least 2% in the study population were retained for the analyses. For the purposes of this study, five impairment domains were considered: mental functions; sensory functions; neuromusculoskeletal and movement-related functions; continence functions; and functions related to prosthetic or orthotic use. A composite category including any impairment was additionally considered. Individuals presenting with multiple impairments were assigned to all relevant categories. To account for co-occurrence, all regression models were adjusted for the presence of each impairment category.

Additional covariates included sociodemographic characteristics (age at diagnosis, sex, marital status, educational level, and area-based deprivation index), clinical characteristics (metastatic status at diagnosis, number of physical comorbidities), and calendar period of diagnosis (pre-COVID-19, 2018–19 and COVID-19, 2020–21). Cancer-related variables and demographic information were obtained from the Milan Cancer Registry, a member of the Italian Association of Cancer Registries and the International Association of Cancer Registries. Socioeconomic status was assessed using an area-level deprivation index derived from Italian census data, based on the revised Caranci index, categorized into quintiles [28]. For the present analyses, the index was dichotomized into two categories: less deprived (quintiles 1–2) and more deprived (quintiles 3–5). Physical comorbidity burden was defined as the number of chronic conditions (0, 1, 2, or ≥3) identified in healthcare records up to 6 months before cancer diagnosis, according to regional definitions [29], including cardiovascular, neurological, genitourinary, cerebrovascular diseases, and type 2 diabetes.

### Statistical analysis

We employed descriptive statistics to examine baseline sociodemographic and clinical characteristics of individuals with different impairment categories. Differences between individuals with and without impairments were assessed using Pearson’s *χ*^2^ test.

Multivariable logistic regression models were fitted to estimate crude and adjusted odds ratios (aORs) with 95% confidence intervals (CIs) for EP versus non-EP. Analyses were first conducted using a binary indicator of any impairment versus none, followed by models including all impairment categories simultaneously. All models were adjusted for age, sex, area-level deprivation, number of comorbidities, metastatic status at diagnosis, and period of diagnosis. Interaction terms between impairment categories and pandemic period were formally tested using likelihood ratio tests. Missing values for variables used only in descriptive analyses were retained and reported as missing where applicable. Multivariable regression analyses were based on complete cases for the variables included in each model.

Overall survival was defined as the time from lung cancer diagnosis to death from any cause, with censoring at the end of follow-up for individuals still alive. Survival analyses were conducted using multivariable Cox proportional hazards models, including all impairment categories simultaneously and adjusting for age, sex, comorbidity burden, metastatic status, and route to diagnosis. Hazard ratios (HRs) with 95% CIs were estimated. Adjusted survival curves were derived using direct standardization over the joint distribution of covariates in the study population and were stratified by metastatic status and RtD. The proportional hazards assumption was evaluated using Schoenfeld residuals.

All statistical tests were two-sided, with a significance threshold set at *P* < .05. Analyses were performed using SAS software, version 9.4.

### Ethical approval

The study protocol was approved by the Ethics Committee “Comitato Etico Territoriale Lombardia 3” (approval number 5328_20.11.2024_P). The requirement for informed consent was waived by the Ethics Committee because the study was based on anonymized routinely collected cancer registry and administrative healthcare data and involved no direct contact with patients.

## Results

### Participant characteristics

Between 2018 and 2021, 9285 individuals were diagnosed with lung cancer in the provinces of Milan and Lodi. Among them, 20.7% (*n* = 1924) had at least one pre-existing impairment. Specifically, 7.2% of individuals had impairments of mental functions, 2.2% of sensory functions, 4.2% of neuromusculoskeletal and movement-related functions, 4.0% of continence functions and 11.4% impairments related to prosthetic or orthotic use (Table 1). Overall, patients with any impairment were slightly older than those without impairments, with median ages of 77 years (interquartile range [IQR]: 69–83) and 73 years (IQR: 65–79), respectively. Median age at diagnosis varied across impairment categories: individuals with continence-related impairments had the highest median age (81 years; IQR: 75–87), followed by those with sensory impairments (80 years; IQR: 75–86), and by individuals with impairments of mental functions and those with impairments related to prosthetic or orthotic use (78 years; IQR: 70–84), while individuals with neuromusculoskeletal and movement-related impairments had the lowest median age (75 years; IQR: 67–81). The lower median age observed for the composite any-impairment group compared with the individual impairment domains reflects the broader and more heterogeneous age distribution of the composite group, which includes individuals across different, potentially overlapping impairment domains.

**Table 1:** Characteristics of lung cancer patients with and without pre-existing impairments (incidence years 2018–21).

	Mental functions			Sensory functions			Neuromusculoskeletal and movement-related functions			Support with incontinence aids			Use of major prosthetic devices			Any impairment		
	No*N* = 861892.8%	Yes*N* = 6677.2%	*P-*value	No*N* = 908397.8%	Yes*N* = 2022.2%	*P*-value	No*N* = 889695.8%	Yes*N* = 3894.2%	*P*-value	No*N* = 891796.0%	Yes*N* = 3684.0%	*P-*value	No*N* = 823188.7%	Yes*N* = 105411.4%	*P-*value	No*N* = 736179.3%	Yes*N* = 192420.7%	*P*-value
**Age**			<.001			<.001			.115			<.001			<.001			<.001
** <50**	220	10		228	2		223	7		230	0		216	14		206	24	
	2.6%	1.5%		2.5%	1.0%		2.5%	1.8%		2.6%	0,0%		2.6%	1.3%		2.8%	1.3%	
**50–59**	827	52		878	1		839	40		869	10		829	50		746	133	
	9.6%	7.8%		9.7%	0.5%		9.4%	10.3%		9.8%	2.7%		10.1%	4.7%		10.1%	6.9%	
**60–69**	2023	102		2106	19		2049	76		2086	39		1944	181		1796	329	
	23.5%	15.3%		23.2%	9.4%		23.0%	19.5%		23.4%	10.6%		23.6%	17.2%		24.4%	17.1%	
**70–79**	3248	205		3376	77		3306	147		3344	109		3111	342		2806	647	
	37.7%	30.7%		37.2%	38.1%		37.2%	37.8%		37.5%	29.6%		37.8%	32.5%		38.1%	33.6%	
**80–89**	2032	237		2188	81		2158	111		2110	159		1890	379		1621	648	
	23.6%	35.5%		24.1%	40.1%		24.3%	28,5%		23.7%	43.2%		23.0%	36.0%		22.0%	33.7%	
**≥90**	268	61		307	22		321	8		278	51		241	88		186	143	
	3.1%	9.2%		3.4%	10.9%		3.6%	2.1%		3.1%	13.9%		2.9%	8.4%		2.5%	7.4%	
**Gender**			.121			.525			.661			.688			.769			.973
**Male**	5.467	403		5.738	132		5.620	250		5.641	229		5.208	662		4.653	1.217	
	63.4%	60.4%		63.2%	65.4%		63.2%	64.3%		63.3%	62.2%		63.3%	62.8%		63.2%	63.3%	
**Female**	3.151	264		3.345	70		3.276	139		3.276	139		3.023	392		2.708	707	
	36.6%	39.6%		36.8%	34.7%		36.8%	35.7%		36.7%	37.8%		36.7%	37.2%		36.8%	36.8%	
**Deprivation index quintile^a^**			.098			.687			.982			.722			.613			.090
**1–2**	3180	226		3329	77		3261	145		3275	131		3020	386		2728	678	
	38.1%	34.9%		37.9%	39.3%		37.9%	38.0%		37.9%	37.0%		38.0%	37.2%		38.4%	36.2%	
**3–5**	5158	422		5461	119		5343	237		5357	223		4928	652		4386	1194	
	61.9%	65.1%		62.1%	60.7%		62.1%	62.0%		62.1%	63.0%		62.0%	62.8%		61.7%	63.8%	
**Marital status**			<.001			<.001			.059			<.001			<.001			<.001
**Single**	1.141	125		1.251	15		1.197	69		1.223	43		1.160	106		1.011	255	
	13.4%	18.8%		13.9%	7.4%		13.6%	17.8%		13.8%	11.7%		14.2%	10.1%		13.9%	13.3%	
**Married**	5.442	334		5.659	117		5.534	242		5.579	197		5.130	646		4.656	1.120	
	63.7%	50.3%		62.9%	57.9%		62.8%	62.4%		63.1%	53.7%		62.9%	61.4%		63.9%	58.4%	
**Widowed**	1503	178		1617	64		1616	65		1565	116		1405	276		1207	474	
	17.6%	26.8%		18.0%	31.7%		18.3%	16.8%		17.7%	31.6%		17.2%	26.2%		16.6%	24.7%	
**Divorced**	182	16		194	4		193	5		191	7		187	11		164	34	
	2.1%	2.4%		2.2%	2.0%		2.2%	1.3%		2.2%	1.9%		2.3%	1.1%		2.3%	1.8%	
**Educational level**			<.001			.009			<.001			<.001			<.001			<.001
**None/primary school**	1.773	177		1.890	60		1.877	73		1.852	98		1.664	286		1.470	480	
	20.6%	26.5%		20.8%	29.7%		21.1%	18.8%		20.8%	26.6%		20.2%	27.1%		20.0%	25.0%	
**Secondary school**	5.071	384		5.341	114		5.195	260		5.225	230		4.814	641		4.300	1.155	
	58.8%	57.6%		58.8%	56.4%		58.4%	66.8%		58.6%	62.5%		58.5%	60.8%		58.4%	60.0%	
**Diploma/degree/PhD**	770	52		811	11		781	41		800	22		752	70		669	153	
	8.9%	7.8%		8.9%	5.5%		8.8%	10.5%		9.0%	6.0%		9.1%	6.6%		9.1%	8.0%	
**Number of chronic conditions**			<.001			<.001			<.001			<.001			<.001			<.001
**0**	4.378	239		4.558	59		4.494	123		4.504	113		4.265	352		3.923	694	
	50.8%	35.8%		50.2%	29.2%		50.5%	31.6%		50.5%	30.7%		51.8%	33.4%		53.3%	36.1%	
**1**	2.512	205		2.646	71		2.610	107		2.602	115		2.404	313		2.123	594	
	29.2%	30.7%		29.1%	35.2%		29.3%	27.5%		29.2%	31.3%		29.2%	29.7%		28.8%	30.9%	
**2**	1.198	120		1268	50		1234	84		1237	81		1077	241		942	376	
	13.9%	18.0%		14.0%	24.8%		13.9%	21.6%		13.9%	22.0%		13.1%	22.9%		12.8%	19.5%	
**3+**	530	103		611	22		558	75		574	59		485	148		373	260	
	6.2%	15.4%		6.7%	10.9%		6.3%	19.3%		6.4%	16.0%		5.9%	14.0%		5.1%	13.5%	
**Diagnostic pathway**			<.001			.840			<.001			.003			<.001			<.001
**EP**	3.246	361		3.529	78		3.405	202		3.438	169		3.073	534		2.650	957	
	38.3%	55.3%		39.5%	38.8%		38.9%	52.9%		39.2%	47.2%		38.0%	51.5%		36.6%	50.6%	
**IP/OP**	5.234	292		5.403	123		5.346	180		5.337	189		5.023	503		4.590	936	
	61.7%	44.7%		60.5%	61.2%		61.1%	47.1%		60.8%	52.8%		62.0%	48.5%		63.4%	49.5%	
**Stage at diagnosis**			.318			<.001			<.001			<.001			.771			.223
**Metastatic**	3072	225		3248	49		3121	176		3196	101		2927	370		2591	706	
	35.7%	33.7%		35.8%	24.3%		35.1%	45.2%		35.8%	27.5%		35.6%	35.1%		35.2%	36.7%	
**Non metastatic**	5546	442		5835	153		5775	213		5721	267		5304	684		4770	1218	
	64.4%	66.3%		64.2%	75.7%		64.9%	54.8%		64.2%	72.6%		64.4%	64.9%		64.8%	63.3%	

a Higher quintiles correspond to higher levels of deprivation, so that quintile 1 is the least deprived.

Abbreviations: EP, emergency presentation; IP, inpatient; OP, outpatient.

Individuals with impairments, compared with those without, were more likely to be widowed, have lower educational attainment and have a higher prevalence of two or more chronic conditions. EP was significantly more frequent among individuals with impairments than among those without, except for sensory impairments, ranging from 55.3% among individuals with mental impairments to 47.2% among those with continence-related impairments, compared with 36.6% among individuals without impairments (all *P* < 0.05; Table 1).

### Multivariable analyses estimating the likelihood of emergency cancer diagnosis by functional impairments and patient characteristics

In the multivariable logistic regression analysis (Fig. 1), several impairment domains and clinical characteristics were independently associated with the odds of EP. Overall, the presence of any pre-existing impairment was associated with higher odds of EP compared with no impairment (aOR = 1.64, 95% CI 1.48–1.83). Individuals with impairment of mental functions had significantly increased odds of EP, compared to individuals without such impairment (aOR = 1.67, 95% CI 1.41–1.98), as were neuromusculoskeletal impairments (aOR = 1.29, 95% CI 1.04–1.61) and the use of major prosthetic devices (aOR = 1.50, 95% CI 1.29–1.75). By contrast, impairment of sensory functions was associated with lower odds of EP (aOR = 0.69, 95% CI 0.50–0.94). No statistically significant association was observed for the use of incontinence aids (aOR = 0.99, 95% CI 0.78–1.25).

**Figure 1:**
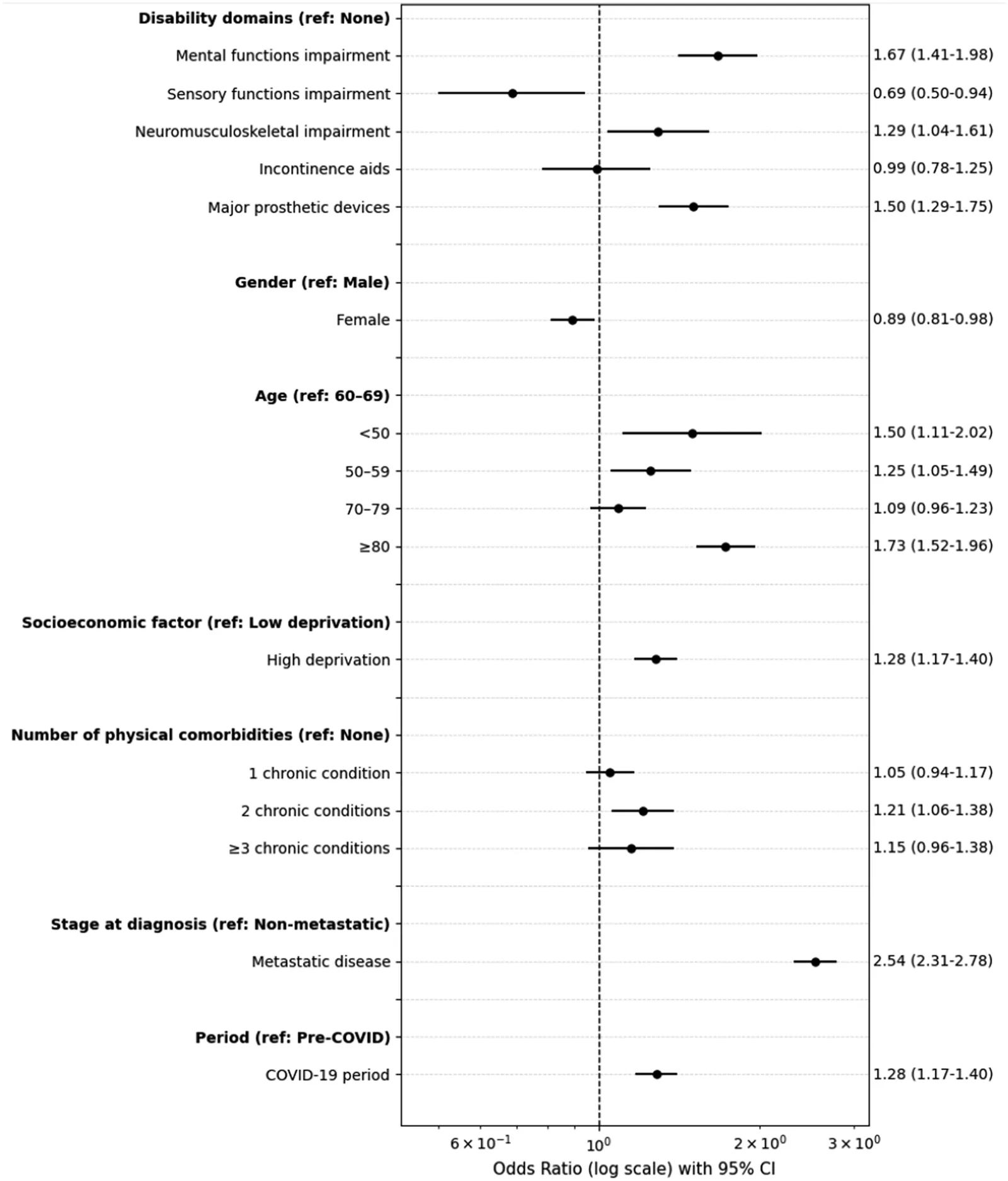
Multivariable logistic regression assessing the odds of cancer diagnosis following an EP by ICF-based impairment categories (mental, sensory, neuromusculoskeletal and movement-related, continence-related, prosthetic or orthotic-related), adjusting for sociodemographic characteristics, number of comorbidities, metastatic status and period of diagnosis (pre-COVID-19, 2018–19 and COVID-19, 2020–21), among patients with lung cancer diagnosed in 2018–21.

Female versus male sex was associated with lower odds of EP (aOR 0.89, 95% CI 0.81–0.98). Patients aged <50 years (aOR = 1.50, 95% CI 1.11–2.02), 50–59 years (aOR = 1.25, 95% CI 1.05–1.49), and ≥80 years (aOR = 1.73, 95% CI 1.52–1.96) had higher odds of EP compared with the reference group aged 60–69. Socioeconomic deprivation was associated with higher odds of EP (aOR = 1.28, 95% CI 1.17–1.40). Having two chronic conditions versus none was associated with significantly higher odds of EP (aOR = 1.21, 95% CI 1.06–1.38). Metastatic disease at diagnosis was associated with a more than twofold increased odds of EP compared with non-metastatic disease (aOR = 2.54, 95% CI 2.31–2.78).

Finally, diagnosis during the COVID-19 period was associated with higher odds of EP compared with the pre-COVID-19 period (aOR = 1.28, 95% CI 1.17–1.40). Period-stratified models yielded similar findings, with somewhat stronger associations during the COVID-19 period for impairments of mental functions (aOR = 1.77, 95% CI 1.39–2.26), impairments related to prosthetic or orthotic use (aOR = 1.60, 95% CI 1.30–1.98), and neuromusculoskeletal impairments (aOR = 1.43, 95% CI 1.03–1.98) (Supplementary Table S1).

### One-year survival by impairment status, overall and stratified by cancer metastatic status and diagnostic route

In the fully adjusted multivariable Cox proportional hazards model, several impairment domains were independently associated with 1-year all-cause mortality. People with impairment of mental functions had a significantly higher hazard of death, compared to people without such impairment (HR = 1.18, 95% CI 1.08–1.30). Similarly, the presence of major prosthetic or orthotic devices was associated with increased hazard of death (HR = 1.22, 95% CI 1.13–1.32). In contrast, impairment of sensory functions was associated with a lower hazard of death (HR = 0.81, 95% CI 0.68–0.95). No significant associations were observed for neuromusculoskeletal and movement-related impairments (HR = 1.05, 95% CI 0.94–1.18) or for the use of incontinence aids (HR = 1.08, 95% CI 0.96–1.21).

Stratified analyses by metastatic status at diagnosis revealed heterogeneity in the associations between impairment status and survival, after adjustment for potential confounders (Supplementary Table S2). Among patients with metastatic cancer at diagnosis, impairment status did not affect 1-year survival across most domains. Only neuromusculoskeletal and movement-related impairments were associated with a significantly increased hazard of death at 1 year (HR = 1.20, 95% CI 1.02–1.41). Among patients with non-metastatic cancer, impairment of mental functions (HR = 1.21, 95% CI 1.08–1.36) and the presence of major prosthetic or orthotic devices (HR = 1.40, 95% CI 1.26–1.55) were associated with an increased hazard of death. Conversely, impairment of sensory functions was associated with reduced hazard of death (HR = 0.80, 95% CI 0.65–0.98). No significant associations were observed for the remaining impairment domains. Consistent with these findings, adjusted survival curves (Fig. 2) showed uniformly poor survival among patients with metastatic disease, with minimal separation between impairment categories, whereas among patients with non-metastatic disease, modest but discernible differences in survival trajectories were observed across impairment domains.

**Figure 2:**
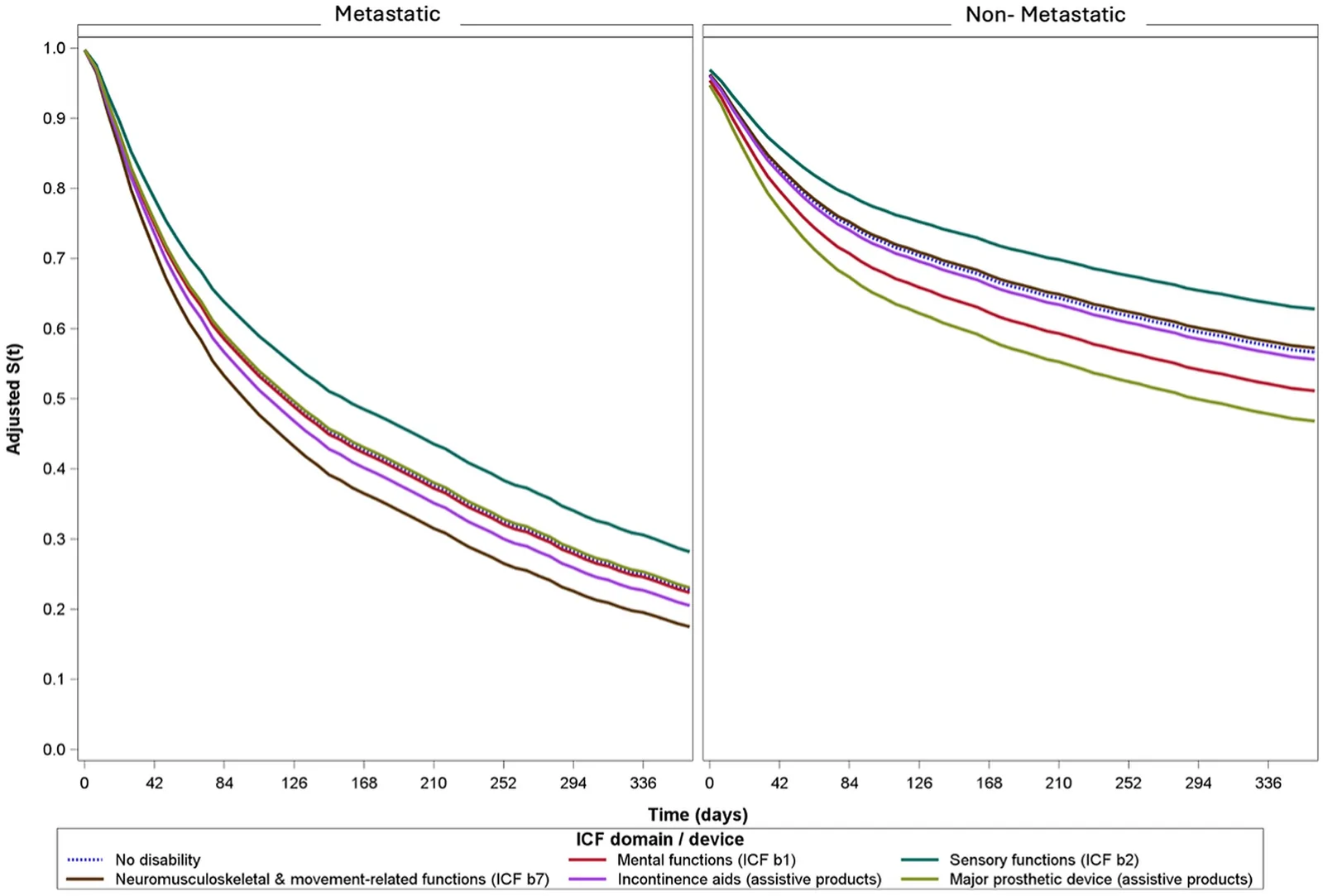
Adjusted 1-year survival curves stratified for metastatic and non-metastatic status at diagnosis, by ICF-based impairment categories (mental functions, sensory functions and pain, neuromusculoskeletal and movement-related functions, continence functions, and prosthetic or orthotic use), compared with patients without such impairments, and adjusted for sex, age group, number of chronic conditions, diagnostic route (emergency presentation versus inpatient/outpatient visits), and period (pre-COVID-19, 2018–19 and COVID-19, 2020–21) (years of incidence 2018–21).

Differences by impairment status were also evident when survival was examined stratified by diagnostic route, after adjustment for potential confounders (Supplementary Table S3). Among patients diagnosed through the EP route, no significant differences in 1-year mortality were observed between patients with and without impairments. Consistently, Fig. 3 shows distinct survival curves by impairment status among non-EP patients, while survival trajectories largely overlap among patients diagnosed through EP. Among patients diagnosed through non-EP routes, impairment related to prosthetic or orthotic use (HR = 1.43, 95% CI 1.28–1.61) and impairment of mental functions (HR = 1.42, 95% CI 1.24–1.63) were associated with a higher 1-year mortality compared with patients without such impairments. In contrast, impairment of sensory functions was associated with a lower hazard of death (HR = 0.77, 95% CI 0.61–0.97).

**Figure 3:**
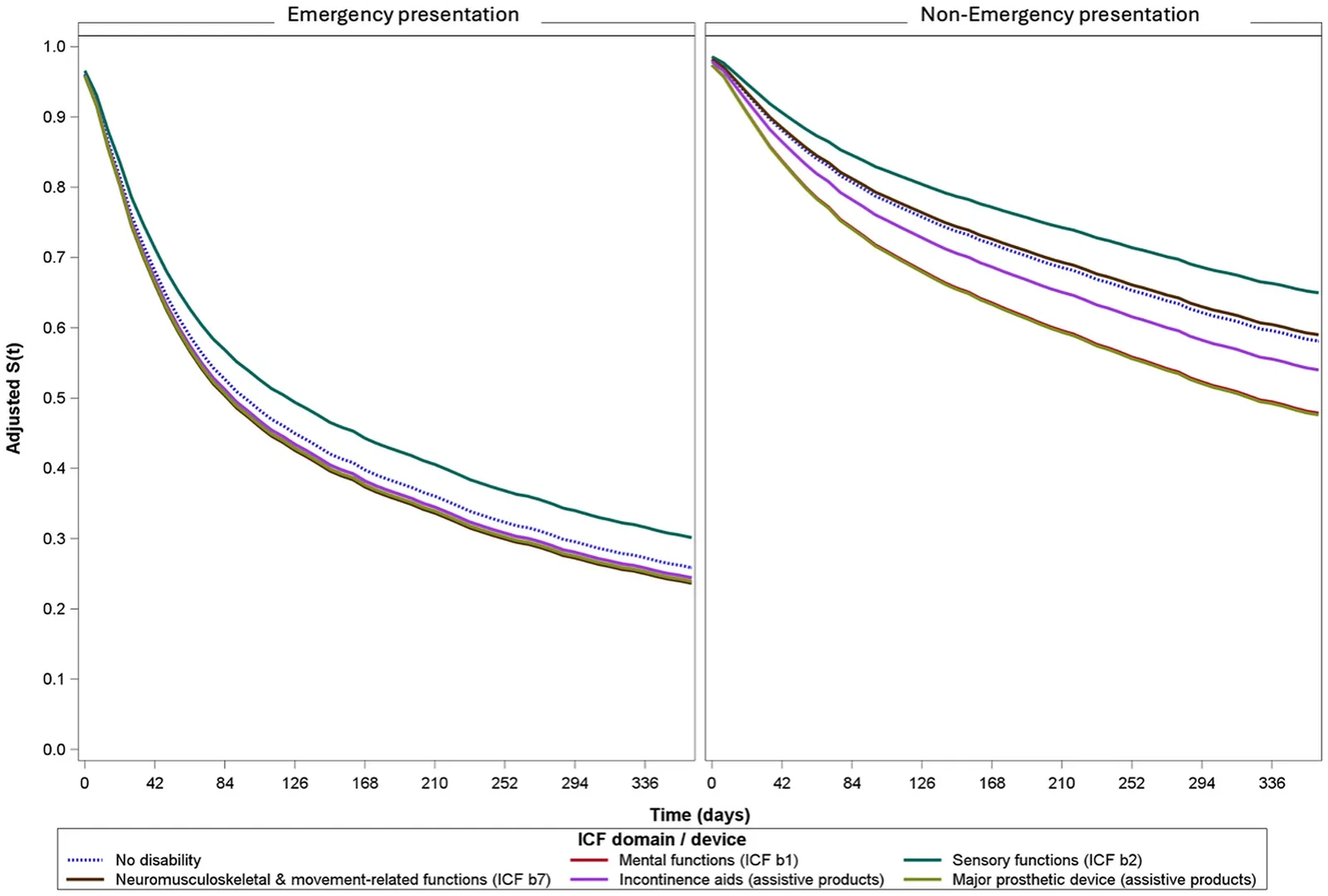
Adjusted 1-year survival curves stratified for emergency presentation and non-emergency diagnostic routes, by ICFs-based impairment categories (mental functions, sensory functions and pain, neuromusculoskeletal and movement-related functions, continence functions, and prosthetic or orthotic use), compared with patients without such impairments, and adjusted for sex, age group, number of chronic conditions, period (pre-COVID-19, 2018–19 and COVID-19, 2020–21), and metastatic status at diagnosis (years of incidence 2018–21).

## Discussion

### Summary of key findings

Patients with pre-existing impairment had 64% higher odds of EP compared with those without impairment, with odds at least 50% higher among those with impairments of mental functions or major prosthetic/orthotic device use. These groups also showed higher 1-year mortality, particularly among non-metastatic cancers and non-EP routes. These findings highlight the need to integrate disability, especially mental and selected physical impairments, into cancer early diagnosis strategies and care pathways to reduce avoidable EPs and improve equity in outcomes.

### Interpretation and comparison with existing literature

Our findings indicate that pre-existing disability represents an independent factor associated with an increased risk of emergency lung cancer diagnosis and poor survival. The magnitude of the adjusted associations observed for specific impairment domains was comparable to or higher than that reported for established sociodemographic risk factors in prior international studies [14].

Population-based analyses of lung cancer routes to diagnosis have established EP as a frequent and prognostically adverse pathway, particularly among populations characterized by social disadvantage, advanced age, and high comorbidity burden [14, 30, 31]. However, pre-existing disability has rarely been incorporated as a structured exposure in studies of EP, and when considered it has typically been operationalized using non-specific or binary indicators. This methodological limitation has constrained the characterization of domain-specific functional impairment contributions to inequalities in lung cancer diagnostic pathways beyond established sociodemographic and clinical determinants. By operationalizing disability through an ICF-based, domain-specific functional impairment framework, the present study provides population-level evidence on disability-related differentials, with some impairments being associated with a particularly higher risk of emergency lung cancer diagnosis.

Although examination of the diagnostic route has been limited, population-based evidence has documented disability-related inequalities in lung cancer staging, treatment, and survival. A nationwide cohort study from South Korea [11] demonstrated higher proportions of unknown stage at diagnosis and substantially lower likelihood of receiving surgery, chemotherapy, or radiotherapy among patients with disabilities, particularly those with severe communication or cognitive impairments. Evidence from the United States [32] further supports this. Analyses of linked SEER–Medicare data indicate that lung cancer patients with disabilities experience lower treatment intensity and shorter survival, consistent with more advanced presentation and diagnostic delay. Although EP was not assessed, this pattern of incomplete diagnostic characterization and treatment underutilization is concordant with our findings and supports the hypothesis that disabilities, and mental or cognitive impairments in particular, can interfere with timely diagnostic access and continuity of care.

In contrast to patterns observed for several other impairment groups, sensory disability was associated with a lower likelihood of cancer diagnosis following an EP. This apparently protective association may reflect differences in healthcare engagement and support structures among individuals with sensory disabilities. One plausible mechanism is greater reliance on accompaniment during healthcare encounters. A nationally representative analysis [33] showed that accompaniment to physician visits was substantially more common among beneficiaries with sensory impairment than among those without (43.46% in hearing impairment, 65.52% in vision impairment, and 72.11% in dual sensory impairment versus 32.59% with no sensory impairment) and was strongly associated with vision impairment and dual sensory impairment after adjustment. Increased accompaniment may facilitate communication, care coordination, and adherence to planned diagnostic pathways, thereby reducing reliance on emergency routes. More broadly, navigation resources, including those provided or facilitated by patient associations and advocacy organizations, have been shown to improve care processes [34]. However, to our knowledge, no published studies have examined emergency-route cancer diagnosis stratified by sensory impairment, precluding direct comparison with our findings. Future research should explicitly assess emergency diagnosis across distinct sensory impairment domains and investigate potential mediating mechanisms such as continuity of care, accessibility accommodations, social support, and navigation.

Consistent with the strong associations observed for impairments of mental functions in our study, a more established body of evidence has specifically examined mental health and neurocognitive conditions in relation to delayed cancer diagnosis [19]. Population-based and registry-linked studies focusing on psychiatric disorders and dementia have documented systematic barriers to timely diagnostic investigation following the presentation of red-flag cancer symptoms, including difficulties in symptom recognition, appraisal, and communication, as well as challenges in navigating diagnostic pathways [15, 35]. Cognitive decline and memory impairment further contribute to delayed help-seeking and diagnostic follow-up, resulting in a higher likelihood of cancer diagnosis following acute complications or incidental findings rather than through planned diagnostic routes [36]. Except for advanced age, dementia was identified as the most significant individual risk factor for EP in England, surpassing several other comorbidities and sociodemographic factors that were examined [37].

Survival differed markedly by diagnostic route and metastatic status. EP and metastatic disease were each associated with substantially poorer 1-year survival, confirming EP as a strong adverse prognostic factor independent of stage and other patient characteristics. Among patients diagnosed through non-emergency routes and among those with non-metastatic disease, clear disability-related survival disparities were observed. Accounting for sociodemographic and clinical characteristics, patients with disabilities, particularly those with impairments of mental functions or reliance on major prosthetic or orthotic devices, experienced worse survival than patients without disabilities. This pattern suggests that disability-related inequalities are most evident among patients who would otherwise be expected to have a more favourable prognosis and may reflect differential access to care, treatment, or other unmeasured clinical or system-level factors. Although our administrative definition primarily captures documented functional impairments, the observed inequalities should not be interpreted as consequences of impairment alone. Within the ICF framework, disability arises from the interaction between functional limitations and contextual factors; differences in diagnostic routes may therefore also reflect variation in the accessibility and responsiveness of healthcare services to patients’ functional and support needs.

### Implications for clinical practice, public health, and research

At the level of individual clinical care, these findings highlight the need to identify patients with functional impairments who may be at higher risk of EP and poorer outcomes. In routine practice, structured assessment of functional impairment could support earlier recognition of diagnostic vulnerability, timely referral, closer follow-up, and tailored reasonable adjustments, particularly for patients with mental, neuromusculoskeletal, or device-related impairments [38].

From a public health and health system perspective, reducing emergency cancer diagnoses and lung cancer mortality requires disability-inclusive early diagnosis strategies. Symptom awareness campaigns, screening programmes, and access to planned diagnostic pathways should be designed to meet diverse functional needs, ensuring accessible information, communication formats, and service delivery. Embedding functional impairment indicators into cancer surveillance and quality monitoring systems could help identify population subgroups at increased risk of EP, monitor inequalities, and guide targeted interventions. In parallel, strengthening disability-competent care through workforce training, integrated care pathways, and better coordination between primary care, specialist services, rehabilitation, and social-care may help reduce avoidable EPs and improve equity in cancer outcomes. At the service level, this could include proactive identification of communication, mobility, healthcare-navigation, and carer-support needs, together with reasonable adjustments such as accessible communication, flexible appointment arrangements, and support in navigating diagnostic services. These findings therefore support the inclusion of disability as a key dimension in cancer control policies, resource allocation, and performance monitoring. The effectiveness of these strategies in reducing emergency diagnosis among people with functional impairments should be evaluated prospectively.

Further research should clarify the mechanisms linking functional impairment to diagnostic routes and survival, including symptom appraisal, help-seeking, continuity of care, diagnostic timeliness, and treatment intensity. Longitudinal studies integrating clinical, administrative, and social data, together with standardized impairment-specific measures, will be important to inform and evaluate interventions aimed at reducing disability-related inequalities in cancer diagnosis and outcomes.

### Strengths and limitations

Key strengths of this study include the systematic use of an ICF-based framework, enabling consistent classification of functional impairment domains, and the use of population-based routinely collected data, which supports comparability and replication across settings and cancer cohorts. Another important strength is the development of a reproducible method for classifying diagnostic routes using linked cancer registry and administrative healthcare data. Similar to established approaches such as the English Routes to Diagnosis algorithm [24], this method leverages routinely collected population-based data to strengthen cancer surveillance and identify potential weaknesses in diagnostic pathways. The use of high-quality cancer registry data enhances the reliability and internal validity of the findings, while the large study population provides sufficient power for stratified analyses by metastatic status and route to diagnosis.

Some limitations should also be acknowledged. Disability status was based on administrative indicators of functional impairment, which may not fully capture the severity, complexity, or psychosocial dimensions of disability. Both algorithms have limitations inherent to the use of routinely collected administrative data. The RtD algorithm retrospectively infers diagnostic pathways from healthcare contacts preceding cancer diagnosis and may therefore result in some route misclassification [25]. Similarly, the ICF-based algorithm may under-ascertain milder or undocumented impairments and does not fully capture impairment severity or the broader contextual dimensions of disability; further individual-level clinical validation is warranted [26]. Therefore, some misclassification of diagnostic route cannot be excluded. The lack of information on symptom onset, symptom type, and patient appraisal prevents direct assessment of symptom-related diagnostic delays. In addition, limited data on the use and timing of diagnostic investigations before cancer diagnosis restricts the ability to distinguish patient-, provider-, and system-level contributors to EP. Residual confounding from unmeasured factors such as smoking, alcohol use, social support, and health literacy cannot be excluded. Future studies incorporating detailed information on access to pre-diagnostic investigations could help clarify the mechanisms linking functional impairment to emergency cancer diagnosis.

## Conclusions

This population-based study provides robust evidence that pre-existing disability is independently associated with EP and short-term mortality in lung cancer. Approximately half of patients with a recorded disability were diagnosed following EP, compared with around one-third of patients without disability. The strongest associations were observed for patients with impairments of mental functions and for those with major prosthetic/orthotic devices. These patient groups were also particularly disadvantaged in terms of 1-year mortality, irrespective of clinical and sociodemographic characteristics.

These findings have important implications for cancer control and quality of care. At the population and health system level, integrating domain-specific functional impairment indicators into population-based cancer surveillance and quality monitoring systems can support the identification of patient groups at increased risk of emergency diagnosis and inform healthcare planning. At the individual level, embedding disability-sensitive approaches within early diagnosis initiatives, including proactive risk stratification, tailored diagnostic pathways, and targeted support for vulnerable patients, may reduce avoidable EPs and improve short-term outcomes for people with mental and physical disabilities.

Key points

- Among 9285 lung cancer patients in Northern Italy, 20.7% had a pre-existing impairment; approximately half of them were diagnosed following emergency presentation, compared with one-third of patients without impairment.
- Impairments of mental functions, neuromusculoskeletal functions, and major prosthetic/orthotic device use were each independently associated with higher odds of emergency cancer diagnosis, after adjustment for age, sex, socioeconomic deprivation, comorbidities, and metastatic status.
- One-year mortality was significantly higher among patients with impairments of mental functions and prosthetic/orthotic device use, particularly among those with non-metastatic disease and those diagnosed through non-emergency routes.
- Disability-related inequalities in emergency cancer diagnosis and short-term survival were independent of established sociodemographic and clinical risk factors, underscoring disability as a distinct dimension of cancer care inequity.
- Integrating domain-specific functional impairment indicators into cancer surveillance and early diagnosis strategies is needed to identify high-risk patient groups and reduce avoidable emergency presentations and outcome disparities.

## Supplementary Material

ckag205_Supplementary_Data

## Data Availability

The data supporting the findings of this article are available at an aggregated level from the authors upon reasonable request and with permission of ATS of Milan. Requests to access should be directed to Carlotta Buzzoni, Scientific Manager of the study, email: cbuzzoni@ats-milano.it.

## References

[1] Elmadani M , Mokaya PO, Omer AAA et al Cancer burden in Europe: a systematic analysis of the GLOBOCAN database (2022). BMC Cancer 2025;25:447. 10.1186/s12885-025-13862-1 PMC11905646 40075331

[2] Bray F , Laversanne M, Sung H et al Global cancer statistics 2022: GLOBOCAN estimates of incidence and mortality worldwide for 36 cancers in 185 countries. CA Cancer J Clin 2024;74:229–63. 10.3322/caac.21834 38572751

[3] Associazione Italiana di Oncologia Medica (AIOM) AIRT (AIRTUM), FAPP d’Argento, ONScreening. numeri del Cancro in Italia 2025. Italy, 2025. https://www.aiom.it/wp-content/uploads/2025/12/2025_NDC_web.pdf

[4] Edwards DJ , Sakellariou D, Anstey S. Barriers to, and facilitators of, access to cancer services and experiences of cancer care for adults with a physical disability: a mixed methods systematic review. Disabil Health J 2020;13:100844. 10.1016/j.dhjo.2019.100844 31668781

[5] Council of Ministers. Italian Government. Law 227/2021—Enabling Act on Disability. Rome, Italy, 2021. https://www.gazzettaufficiale.it/eli/id/2021/12/30/21G00254/SG

[6] Mangone L , Marinelli F, Bisceglia I et al The influence of COVID-19 on new lung cancer diagnoses, by stage and treatment, in Northern Italy. Biology (Basel) 2023;12. 10.3390/biology12030390 PMC10045495 36979081

[7] WHO. Global report on health equity for persons with disabilities. https://www.who.int/publications/i/item/9789240063600

[8] WHO Regional Office for Europe. Good practices on disability-inclusive health The WHO European framework to achieve the highest attainable standard of health for people with disabilities. 2025. https://www.who.int/europe/publications/i/item/WHO-EURO-2025-9550-49322-73722

[9] INDICATORI SU DISABILITÀ E FRAGILITÀ NOTA DESCRITTIVA. https://www.istat.it/wp-content/uploads/2023/12/disabilit%C3%A0_pon_dic2023.pdf

[10] Iezzoni LI , Ngo LH, Li D et al Treatment disparities for disabled medicare beneficiaries with stage I non-small cell lung cancer. Arch Phys Med Rehabil 2008;89:595–601. 10.1016/j.apmr.2007.09.042 18373987

[11] Shin DW , Cho JH, Noh JM et al Disparities in the diagnosis and treatment of lung cancer among people with disabilities. J Thoracic Oncol 2019;14:163–75. 10.1016/j.jtho.2018.10.158 30453082

[12] Malapelle U , Pilotto S, Passiglia F et al Dealing with NSCLC EGFR mutation testing and treatment: a comprehensive review with an Italian real-world perspective. Crit Rev Oncol Hematol 2021;160:103300. 10.1016/j.critrevonc.2021.103300 33744362

[13] Associazione Italiana di Oncologia Medica (AIOM) AIRT (AIRTUM), FAPP d’Argento, ONScreening. I numeri del cancro in Italia 2023. Italy, 2023. https://www.aiom.it/wp-content/uploads/2023/12/2023_AIOM_NDC-web.pdf

[14] McPhail S , Swann R, Johnson SA et al; ICBP Module 9 Emergency Presentations Working Group. Risk factors and prognostic implications of diagnosis of cancer within 30 days after an emergency hospital admission (emergency presentation): an International Cancer Benchmarking Partnership (ICBP) population-based study. Lancet Oncol 2022;23:587–600. 10.1016/S1470-2045(22)00127-9 PMC9046095 35397210

[15] Benitez Majano S , Lyratzopoulos G, de Wit NJ et al Mental health morbidities and time to cancer diagnosis among adults with colon cancer in England. JAMA Netw Open 2022;5:e2238569. 10.1001/jamanetworkopen.2022.38569 PMC9623442 36315146

[16] Maringe C , Pashayan N, Rubio FJ et al Trends in lung cancer emergency presentation in England, 2006–2013: is there a pattern by general practice? BMC Cancer 2018;18:615. 10.1186/s12885-018-4476-5 PMC5984417 29855264

[17] Christensen NL , Gouliaev A, McPhail S et al Routes to diagnosis in Danish lung cancer patients: emergency presentation, age and smoking history—a population-based cohort study. Clin Lung Cancer 2024;25: e348–56. 10.1016/j.cllc.2024.05.009. 38890094

[18] Vallome G , Cafaro I, Bottini A et al Diagnosis of lung cancer following emergency admission: examining care pathways, clinical outcomes, and advanced NSCLC treatment in an Italian cancer center. Heliyon 2023;9:e21177. 10.1016/j.heliyon.2023.e21177 PMC10623281 37928020

[19] Cunningham R , Stanley J, Imlach F et al Cancer diagnosis after emergency presentations in people with mental health and substance use conditions: a national cohort study. BMC Cancer 2024;24:546. 10.1186/s12885-024-12292-9 PMC11062004 38689242

[20] Pennisi F , Buzzoni C, Gervasi F et al Emergency colon cancer diagnosis in people with mental health conditions: a population-based cohort study in northern Italy. BMJ Ment Health 2025;28. 10.1136/bmjment-2025-301733 PMC12215114 40592734

[21] Implemented ICF Update Proposals 2018. n.d.

[22] WHO. International Classification of Diseases for Oncology, 3rd Edition (ICD-O-3). 2019. https://www.who.int/standards/classifications/other-classifications/international-classification-of-diseases-for-oncology

[23] von Elm E , Altman DG, Egger M et al; STROBE Initiative. The strengthening the reporting of observational studies in epidemiology (STROBE) statement: guidelines for reporting observational studies. J Clin Epidemiol 2008;61:344–9. 10.1016/j.jclinepi.2007.11.008 18313558

[24] Elliss-Brookes L , McPhail S, Ives A et al Routes to diagnosis for cancer—determining the patient journey using multiple routine data sets. Br J Cancer 2012;107:1220–6. 10.1038/bjc.2012.408 PMC3494426 22996611

[25] Pennisi F , Buzzoni C, Russo AG et al Comorbidities, socioeconomic status, and colorectal cancer diagnostic route. JAMA Netw Open 2025;8:e258867. 10.1001/jamanetworkopen.2025.8867 PMC12056571 40327340

[26] Leonardi M , Lee H, Kostanjsek N et al 20 years of ICF—international classification of functioning, disability and health: uses and applications around the world. Int J Environ Res Public Health 2022;19. 10.3390/ijerph191811321 PMC9517056 36141593

[27] Friedman DJ , Gibson Parrish R, Fox MH. A review of global literature on using administrative data to estimate prevalence of intellectual and developmental disabilities. J Policy Pract Intellect Disabil 2018;15:43–62. 10.1111/jppi.12220 PMC5903294 29681966

[28] Rosano A , Pacelli B, Zengarini N et al Update and review of the 2011 Italian deprivation index calculated at the census section level. Epidemiol Prev 2020;44:162–70. 10.19191/EP20.2-3.P162.039 32631016

[29] Lombardy Region. Deliberazione n. X/ 6164 del 30/07/2017 “Governo della domanda: avvio della presa in carico di pazienti cronici e fragili. Determinazioni in attuazione dell’art. 9 della legge n. 23/2015”. I. 2015. https://www.medicinaepersona.org/wp-content/uploads/2017/02/DGR6164_2017-governo-delladomanda.pdf

[30] Newsom-Davis T. The route to diagnosis: emergency presentation of lung cancer. Lung Cancer Manag 2017;6:67–73. 10.2217/lmt-2017-0004 PMC6310353 30643572

[31] Gurney J , Davies A, Stanley J et al Emergency presentation prior to lung cancer diagnosis: a national-level examination of disparities and survival outcomes. Lung Cancer 2023;179:107174. 10.1016/j.lungcan.2023.03.010 36958240

[32] Chirikos TN , Roetzheim RG, McCarthy EP et al Cost disparities in lung cancer treatment by disability status, sex, and race. Disabil Health J 2008;1:108–15. 10.1016/j.dhjo.2008.01.004 PMC2770703 19881893

[33] Reed NS , Assi L, Pedersen E et al Accompaniment to healthcare visits: the impact of sensory impairment. BMC Health Serv Res 2020;20:990. 10.1186/s12913-020-05829-8 PMC7594990 33121483

[34] Chen M , Wu VS, Falk D et al Patient navigation in cancer treatment: a systematic review. Curr Oncol Rep 2024;26:504–37. 10.1007/s11912-024-01514-9 PMC11063100 38581470

[35] Pennisi F , Ricciardi GE, von Wagner C et al Impact of self-reported long-term mental health morbidity on help-seeking and diagnostic testing for bowel-related cancer symptoms: a vignette study. Cancer Med 2024;13:e70426. 10.1002/cam4.70426 PMC11621967 39641393

[36] McWilliams L , Farrell C, Grande G et al A systematic review of the prevalence of comorbid cancer and dementia and its implications for cancer-related care. Aging Ment Health 2018;22:1254–71. 10.1080/13607863.2017.1348476 28718298

[37] Wallace D , Walker K, Kuryba A et al Identifying patients at risk of emergency admission for colorectal cancer. Br J Cancer 2014;111:577–80. 10.1038/bjc.2014.300 PMC4119975 24921910

[38] Siviş S , Turner A, Little H et al Exploring experiences of reasonable adjustments in cancer care among patients with disabilities and their carers: a focus group study in south west England. J Cancer Policy 2025;46:100641. 10.1016/j.jcpo.2025.100641 40975494

