# Supplementary material for: Disability-related inequalities in lung cancer diagnostic pathways and outcomes: a population-based study in Northern Italy
Source: Eur J Public Health. 2026 Nov 10;36(6):ckag205. doi: 10.1093/eurpub/ckag205 (PMC13750167; doi:10.1093/eurpub/ckag205)
Supplement: Supplementary file 1 [file ckag205_supplementary_data.zip › ejph-2026-05-om-0547-File005.docx]

STROBE Statement Checklist for Cohort Studies

**Manuscript: Disability-related inequalities in lung cancer diagnostic pathways and outcomes: a population-based study in Northern Italy**

| **STROBE item** | **No.** | **Reporting recommendation (concise)** | **Where reported in manuscript** |
| --- | --- | --- | --- |
| Title and abstract | 1a | Identify the observational study design in the title or abstract. | p. 1 (title); p. 2 (Methods in Abstract) |
| Title and abstract | 1b | Provide an informative and balanced abstract summary of methods and findings. | p. 2 (Abstract) |
| Background/rationale | 2 | Explain the scientific background and rationale. | pp. 3–4 (Introduction) |
| Objectives | 3 | State the study objectives and any prespecified hypotheses. | p. 4 (end of Introduction) |
| Study design | 4 | Present the key elements of the study design early in the Methods. | p. 4, Section 2.1 |
| Setting | 5 | Describe setting, locations, and relevant study dates, including follow-up/data collection periods. | p. 4, Section 2.1; p. 6, Section 2.3 |
| Participants | 6a | Describe eligibility criteria, participant selection/data sources, and follow-up. | p. 4, Section 2.1; p. 6, Section 2.3 |
| Participants | 6b | For matched cohorts, report matching criteria and numbers. | Not applicable: this was not a matched cohort study. |
| Variables | 7 | Define outcomes, exposures, predictors/confounders, and relevant diagnostic criteria. | pp. 4–6, Section 2.2 |
| Data sources / measurement | 8 | Describe data sources and measurement/ascertainment methods for variables of interest. | pp. 4–6, Section 2.2; Supplementary Figures S1–S2 |
| Bias | 9 | Describe efforts to address potential sources of bias. | p. 6, Section 2.3; pp. 12–13, Section 4.4 (Strengths and limitations) |
| Study size | 10 | Explain how the study size was determined. | p. 4, Section 2.1: all eligible incident lung cancer cases in the study area and period were included; p. 7 reports the resulting cohort size (n=9,285). |
| Quantitative variables | 11 | Explain handling and categorisation of quantitative variables. | pp. 5–6, Sections 2.2–2.3 (age, deprivation index, comorbidity count, impairment-domain prevalence threshold). |
| Statistical methods | 12a | Describe statistical methods, including methods used to control for confounding. | p. 6, Section 2.3 |
| Statistical methods | 12b | Describe subgroup and interaction analyses. | p. 6, Section 2.3; pp. 8–9, Sections 3.2–3.3; Tables S1–S3 |
| Statistical methods | 12c | Explain how missing data were handled. | p. 6, Section 2.3: descriptive missing values retained/reported as applicable; multivariable models based on complete cases for variables included in each model. |
| Statistical methods | 12d | Explain how loss to follow-up/censoring was handled, if applicable. | p. 6, Section 2.3: survival time, censoring, and one-year follow-up definition. |
| Statistical methods | 12e | Describe sensitivity analyses, if performed. | Not applicable: no separate sensitivity analysis was prespecified/reported. |
| Participants | 13a | Report numbers of individuals at relevant stages of the study and numbers analysed. | p. 7, Section 3.1; Table 1; model-specific analytic samples are determined by complete-case availability. |
| Participants | 13b | Give reasons for non-participation/exclusion at relevant stages. | p. 4, Section 2.1 (population-based eligibility); not otherwise applicable because routinely collected registry data were used. |
| Participants | 13c | Consider presenting a participant flow diagram. | Not presented; the population-based cohort comprised all eligible incident cases identified in the registry. |
| Descriptive data | 14a | Report participant characteristics, exposures, and potential confounders. | p. 7, Section 3.1; Table 1, pp. 18–23 |
| Descriptive data | 14b | Report missing data for variables of interest. | Table 1, where applicable; missing-data handling is described in Section 2.3. |
| Descriptive data | 14c | Summarise follow-up time. | p. 6, Section 2.3: follow-up for survival analyses defined from diagnosis and restricted to one year. |
| Outcome data | 15 | Report outcome events or summary measures over time. | pp. 7–9, Sections 3.1–3.3; Table 1; Figures 2–3; Tables S2–S3 |
| Main results | 16a | Report unadjusted and adjusted estimates with precision and identify adjustment variables. | pp. 7–9, Sections 3.1–3.3; Figure 1; Tables S1–S3 |
| Main results | 16b | Report boundaries used for categorised continuous variables. | pp. 5–6, Sections 2.2–2.3; Table 1 |
| Main results | 16c | Where relevant, consider translating relative estimates into absolute risks. | Not applicable to the primary adjusted effect estimates; descriptive absolute proportions of emergency presentation are reported in Section 3.1 and Table 1. |
| Other analyses | 17 | Report other analyses, including subgroup/interaction analyses. | pp. 8–9, Sections 3.2–3.3; Tables S1–S3; Figures 2–3 |
| Key results | 18 | Summarise key results in relation to study objectives. | p. 10, Section 4.1 |
| Limitations | 19 | Discuss limitations, including potential bias and imprecision. | pp. 12–13, Section 4.4 |
| Interpretation | 20 | Provide a cautious overall interpretation considering objectives, limitations, multiplicity, and other evidence. | pp. 10–13, Sections 4.1–4.4 |
| Generalisability | 21 | Discuss the generalisability/external validity of the findings. | pp. 12–13, Sections 4.3–4.4 |
| Funding | 22 | Report funding and the role of funders. | p. 15, Funding information |

**STROBE reference:** von Elm E, Altman DG, Egger M, et al. The Strengthening the Reporting of Observational Studies in Epidemiology (STROBE) Statement: guidelines for reporting observational studies. The Lancet. 2007;370:1453–1457.

**Checklist source:** STROBE Initiative, cohort-study checklist. Recommendations in this completed table are concise paraphrases for submission use.
