# Supplementary material for: Disability-related inequalities in lung cancer diagnostic pathways and outcomes: a population-based study in Northern Italy
Source: Eur J Public Health. 2026 Nov 10;36(6):ckag205. doi: 10.1093/eurpub/ckag205 (PMC13750167; doi:10.1093/eurpub/ckag205)
Supplement: Supplementary file 1 [file ckag205_supplementary_data.zip › ejph-2026-05-om-0547-File006.docx]

**Table S1.** Multivariable logistic regression assessing the association between impairment categories (mental functions, sensory functions and pain, neuromusculoskeletal and movement-related functions, continence functions and prosthetic or orthotic use), sex, age group, deprivation index, metastatic status and number of chronic conditions and the risk of emergency presentation (EP) among lung cancer patients diagnosed in the pre- COVID-19 (2018–2019) and COVID-19 (2020–2021) periods.

|  | **Emergency presentation (EP)** | |
| --- | --- | --- |
|  | **Pre-** **COVID-19 OR (95% CI)** | **During COVID-19 OR (95% CI)** |
| **Impairment categories** (ref: None) |  |  |
| Impairments of mental functions | 1.59 (1.25–2.03) | 1.77 (1.39–2.26) |
| Impairments of sensory functions | 0.87 (0.56–1.36) | 0.55 (0.35–0.86) |
| Impairments of neuromusculoskeletal and movement-related functions | 1.20 (0.89–1.62) | 1.43 (1.03–1.98) |
| Impairments of continence functions | 1.09 (0.79–1.49) | 0.88 (0.62–1.25) |
| Impairments of functions related to prosthetic or orthotic use | 1.40 (1.13–1.73) | 1.60 (1.30–1.98) |
| **Sex** (ref: Male) |  |  |
| Female | 0.92 (0.81–1.05) | 0.86 (0.76–0.99) |
| **Age** (ref: 60–69) |  |  |
| <50 | 2.07 (1.36–3.15) | 1.09 (0.71–1.68) |
| 50–59 | 1.33 (1.05–1.70) | 1.17 (0.92–1.50) |
| 70–79 | 1.17 (0.99–1.38) | 1.01 (0.85–1.20) |
| ≥80 | 1.75 (1.46–2.08) | 1.74 (1.44–2.09) |
| **Deprivation index** (ref: Low) |  |  |
| High deprivation | 1.27 (1.12–1.45) | 1.29 (1.13–1.47) |
| **Metastatic status** (ref: Non-metastatic) |  |  |
| Metastatic disease | 2.39 (2.11–2.71) | 2.74 (2.39–3.13) |
| **Number of chronic conditions** (ref: None) |  |  |
| 1 | 1.19 (1.03–1.38) | 0.92 (0.79–1.07) |
| 2 | 1.29 (1.07–1.55) | 1.12 (0.92–1.37) |
| ≥3 | 1.21 (0.94–1.55) | 1.09 (0.83–1.42) |
