# Supplementary material for: Disability-related inequalities in lung cancer diagnostic pathways and outcomes: a population-based study in Northern Italy
Source: Eur J Public Health. 2026 Nov 10;36(6):ckag205. doi: 10.1093/eurpub/ckag205 (PMC13750167; doi:10.1093/eurpub/ckag205)
Supplement: Supplementary file 1 [file ckag205_supplementary_data.zip › ejph-2026-05-om-0547-File007.docx]

**Table S2.** Association between International Classification of Functioning, Disability and Health (ICF)-based impairment categories and one-year overall mortality, stratified by cancer metastatic status at diagnosis (non-metastatic vs metastatic), adjusted for gender, age, deprivation index, number of chronic conditions, diagnostic route and COVID-19 period.

| **Metastatic status at diagnosis** | **Adjusted HR (95% CI)** |
| --- | --- |
| **Metastatic** (ref: None impairments) |  |
| Impairments of mental functions | 1.01 (0.88–1.17) |
| Impairments of sensory functions and pain | 0.84 (0.62–1.15) |
| Impairments of neuromusculoskeletal and movement-related functions | 1.20 (1.02–1.41) |
| Impairments of continence functions | 1.08 (0.87–1.33) |
| Impairments related to prosthetic or orthotic use | 0.99 (0.88–1.12) |
| **Non-metastatic** (ref: None impairments) |  |
| Impairments of mental functions | 1.21 (1.08–1.36) |
| Impairments of sensory functions and pain | 0.80 (0.65–0.98) |
| Impairments of neuromusculoskeletal and movement-related functions | 0.98 (0.83–1.16) |
| Impairments of continence functions | 1.04 (0.90–1.20) |
| Impairments related to prosthetic or orthotic use | 1.40 (1.26–1.55) |
