# Supplementary material for: Disability-related inequalities in lung cancer diagnostic pathways and outcomes: a population-based study in Northern Italy
Source: Eur J Public Health. 2026 Nov 10;36(6):ckag205. doi: 10.1093/eurpub/ckag205 (PMC13750167; doi:10.1093/eurpub/ckag205)
Supplement: Supplementary file 1 [file ckag205_supplementary_data.zip › ejph-2026-05-om-0547-File008.docx]

**Table S3.** Association between International Classification of Functioning, Disability and Health (ICF)-based impairment categories and one-year overall mortality, stratified by diagnostic route (emergency vs non-emergency presentation), adjusted for gender, age, deprivation index, number of chronic conditions, metastatic status and COVID-19 period.

| **Diagnostic route** | **Adjusted HR (95% CI)** |
| --- | --- |
| **Non-emergency presentation** (ref: None impairments) |  |
| Impairments of mental functions | 1.42 (1.24–1.63) |
| Impairments of sensory functions and pain | 0.77 (0.61–0.97) |
| Impairments of neuromusculoskeletal and movement-related functions | 0.97 (0.81–1.16) |
| Impairments of continence functions | 1.16 (0.98–1.37) |
| Impairments related to prosthetic or orthotic use | 1.43 (1.28–1.61) |
| **Emergency presentation** (ref: None impairments) |  |
| Impairments of mental functions | 1.05 (0.93–1.18) |
| Impairments of sensory functions and pain | 0.88 (0.69–1.12) |
| Impairments of neuromusculoskeletal and movement-related functions | 1.07 (0.92–1.25) |
| Impairments of continence functions | 1.05 (0.88–1.24) |
| Impairments related to prosthetic or orthotic use | 1.07 (0.96–1.19) |
